# Association between hyperglycemia in middle and late pregnancy and maternal-fetal outcomes: a retrospective study

**DOI:** 10.1186/1471-2393-14-34

**Published:** 2014-01-20

**Authors:** Juan Gui, Aizhen Li, Xiaoling Su, Ling Feng

**Affiliations:** 1 Department of Obstetrics and Gynecology, Tongji Hospital, Tongji medical college, Huazhong University of Science and Technology, Wuhan, Hubei, China

## Abstract

**Background:**

The purposes of this study were to explore whether the maternal-fetal outcomes differed among various types of hyperglycemia during pregnancy and whether the values of glycemic screening in the middle phase of pregnancy could predict maternal-fetal outcomes.

**Methods:**

A retrospective study was conducted to study the incidence of maternal-fetal outcomes in 383 singleton pregnant women with diabetes or gestational diabetes admitted to our hospital from November 2007 to March 2013. Patients were divided into three groups: DM (Type 1 and Type 2 diabetes mellitus) group, mGDM (mild gestational diabetes mellitus) group and sGDM (severe gestational diabetes mellitus) group. Maternal basic characteristics, results of oral glucose tolerance test (OGTT), antenatal random glycemia and maternal-fetal outcomes were collected. Binary logistic regression was used to estimate the association of blood glucose with the maternal-fetal outcomes. Predictive accuracy was assessed by calculating the areas under the receiver operating characteristic curves.

**Results:**

The maternal basic characteristics, maternal complications and neonatal complications did not differ significantly between DM group and sGDM group, except neonatal intensive care units admission (NICU). Incidences of preterm, NICU and preeclampsia were significantly lower in the mGDM group than in the DM and sGDM groups (*P* < 0.05). After adjusted by confounding factors, the value of OGTT 0 h could predict pregnancy induced hypertension (PIH) (OR = 1.24, 95% CI [1.04 to 1.46], *P* = 0.015), preterm birth (OR = 1.23, 95% CI [1.03 to 1.47], *P* = 0.025) and stillbirth (OR = 1.55, 95% CI [1.14 to 2.10], *P* = 0.005); antenatal random glycemia could predict preterm birth (OR = 1.19, 95% CI [1.08 to 1.31], *P* < 0.001) and stillbirth (OR = 1.41, 95% CI [1.17 to 1.71], *P* < 0.001).

**Conclusions:**

Pregnant women in the mGDM group have better outcomes than those in the DM and sGDM groups. The values of OGTT in the middle phase of pregnancy and antenatal random glycemia could predict PIH, preterm birth or stillbirth to some extent.

## Background

In recent years, the incidence of gestational diabetes mellitus (GDM) has increased in China. According to the results of a prospective study enrolling more than 10,000 pregnant women in 18 cities, the incidence ranges from 4.3% to 5.1% in China [1,2]. Lots of studies have shown that maternal hyperglycemia during pregnancy is associated with increased risk of specific maternal-fetal complications, including pregnancy induced hypertension (PIH), preeclampsia, cesarean section, stillbirth, congenital defects, neonatal hypoglycemia and neonatal hyperbilirubinemia [3-5]. In the long term, for the mothers, there is an increased risk for developing Type 2 diabetes mellitus (T2DM) after pregnancy [2,6]; for the offspring, studies have provided substantial evidences that intrauterine exposure to maternal hyperglycemia has lifelong effects, including increased risk of obesity [7,8], T2DM [9,10], metabolic [11-14] and cardiovascular disease [15,16] and even cancer [17]. These hyperglycemia-related short or long term dysfunctions are not only confined to women with Type 1 diabetes mellitus (T1DM) or T2DM diagnosed before gestation, but are also observed in women with GDM. Fortunately, studies have shown that appropriate managements such as dietary, moderate exercise, oral hypoglycemic agents and insulin therapy could reduce the risk of complications and improve maternal and neonatal outcomes [18-22]. Therefore, the diagnosis and management of GDM are important in that it poses risks to both the mothers and their babies [6,23].

GDM has been defined as any degree of glucose intolerance with onset or first recognition during pregnancy [24]. If the fasting glucose ≥ 7 mmol/L or HbA1c ≥6.5% or oral glucose tolerance test (OGTT) 2 h ≥ 11.1 mmol/L, one should be considered to be pre-diabetes [24]. The management of this kind of severe gestational diabetes mellitus (sGDM) might be more demanding than mild gestational diabetes mellitus (mGDM) [22]. McCance DR suggested treating and following up sGDM as pre-existing diabetes [25]. However, until recently, it is not clear that whether differences exist in maternal-fetal outcomes among various types of hyperglycemia and whether the values of glycemic screening in the middle phase of pregnancy could predict specific maternal-fetal outcomes. So the aims of our study were to explore that 1) whether the maternal-neonatal outcomes differed among various types of hyperglycemia during pregnancy; 2) whether the values of OGTT and antenatal random glycemia could predict specific maternal or neonatal complications.

## Methods

### Study subjects

This was a retrospective study of 383 singleton pregnant women with pre-existing diabetes mellitus (DM) or GDM and their offspring who delivered in Tongji Hospital affiliated to Huazhong University of Science and Technology from November 2007 to March 2013. The Ethics Committee of Tongji Hospital approved the study (in accordance with the Helsinki declaration). There were 53 pregnant women with DM and 330 with GDM. The inclusion criteria for patients with GDM before November 2011 were as follows [26]: fasting glycemic level ≥ 5.8 mmol/L for at least twice; two or more glycemic values exceed the cutoff points of 75 g OGTT (fasting ≥ 5.6 mmol/L, 1 h ≥ 10.3 mmol/L, 2 h ≥ 8.6 mmol/L, 3 h ≥ 6.7 mmol/L). Others were diagnosed according to the new criteria of American Diabetes Association published in 2011: any of the plasma glucose values exceeded the cutoff points of 75 g OGTT (fasting ≥ 5.1 mmol/L, 1 h ≥ 10.0 mmol/L, 2 h ≥ 8.5 mmol/L) [27]. Patients with the following conditions were excluded from the study: patients with DM had no exact diagnosis and patients with GDM were without definite outcomes of OGTT.

### Groups

Patients with diabetes mellitus diagnosed before gestation were classified in the DM group (n = 53, 2 are T1DM, others are T2DM). For the GDM women, classification was based on values of OGTT. They were divided into two groups including severe GDM (sGDM, n = 135, fasting ≥ 7 mmol/L or OGTT 2 h ≥ 11.1 mmol/L) and mild GDM (mGDM, n = 195, fasting < 7 mmol/L and OGTT 2 h < 11.1 mmol/L).

### Statistical analysis

The data were summarized as frequencies or percentages for categorical variables and as means and standard deviations or medians and interquartile ranges for continuous variables, depending on the distribution. Differences between the groups were compared by the chi-square or Fisher’s exact test for categorical variables and multiple comparisons in ANOV or Kruskal Wallis Test for continuous variables. Associations of blood glucose levels with the maternal and neonatal outcomes were assessed by binary logistic regression, unadjusted first, and then adjusted for age, history of spontaneous abortion, history of stillbirth, family history, living place, diagnose time of GDM, maternal weight before delivery and treatment. Predictive accuracy of maternal/neonatal outcomes and blood sugar levels were assessed by calculating the areas under the receiver operating characteristic (ROC) curves, which were compared according to the method of Hanley and McNeil; optimal cutoff values were chosen as the point on the ROC curve, closest to the top left corner. A nominal 2-sided probability value < 0.05 was considered to indicate statistical significance, and adjustments were made for multiple comparisons in chi-square test and Kruskal Wallis Test (*P*_adjust_ < 0.017). All of the calculations were performed using the SPSS 12.0 (SPSS Inc. Chicago, IL).

## Results

In this study, patients in DM group had a median diabetic history of about 2 years (1.75-5y). The median diagnosis time of GDM was 210 days (186-237d) for mGDM and 233 days (197-253d) for sGDM after pregnancy. The diagnosis time of patients in mGDM group was much earlier than that in sGDM group (*P* = 0.001). There was no significant difference between DM group and sGDM group in maternal basic characteristics, maternal complications and neonatal complications except neonatal intensive care units (NICU) admission. When compared with the DM and sGDM groups, the time of delivery was much later in mGDM group; values of OGTT 0 h, 1 h, 2 h, antenatal random glycemia, incidence of preterm and NICU admission were significantly lower in mGDM group. Significant difference was found in the incidences of PIH and stillbirth only between mGDM and sGDM groups. Family history and history of stillbirth were significantly less in mGDM group than in DM group (*P* < 0.05, *P*_adjust_ < 0.017). Several maternal and neonatal characteristics were similar across the three groups. These details were shown in Table 1.

**Table 1:** Comparison of maternal basic characters, maternal-fetal complications

**Characteristic**	**DM**	**mGDM**	**sGDM**
	**(n = 53)**	**(n = 198)**	**(n = 132)**
Age (year)	30.32 ± 4.62	30.83 ± 4	31 ± 4.76
Family history (%)	26.4	11.6^a^	18.2
History of spontaneous abortion (%)	15.1	14.6	15.2
History of stillbirth (%)	9.4	1.5^a^	4.5
Chronic hypertension (%)	5.7	1	0.8
OGTT 0 h (mmol/L)†	8.5 (7.03-10.42)	5.28 (4.88-5.62)^ab^	7.53 (6.46-9.16)
OGTT 1 h (mmol/L)‡	15.17 ± 1.63	10.64 ± 1.57^ab^	13.35 ± 2.87
OGTT 2 h (mmol/L)§	15.33 ± 3.13	9.12 ± 1.06^ab^	14.13 ± 3.66
Maternal weight before delivery (kg)¶	75 (65.23-89.63)	74 (66.62-80)	73.75 (63–83.5)
Neonatal weight (kg)||	3.49 ± 0.75	3.41 ± 0.46	3.46 ± 0.60
Cesarean rate (%)	88.7	92.9	85.6
Time of delivery (day)	264 (250.5-270)	268 (263–274)^ab^	263.5 (252.25-272)
Antenatal random glycemia (mmol/L)††	7.67 (5.5-10.22)	5.61 (4.75-6.7)^ab^	7.31 (5.17-9.63)
Ketoacidosis (%)	0	0	1.5
PIH (%)	18.9	9.1^b^	27.3
Preeclampsia (%)	15.1	5.6^b^	16.7
Premature rupture of membranes (%)	17	6.1	8.3
Fetal distress (%)	13.2	4	7.6
Polyhydramnios (%)	7.5	2	2.3
Oligohydramnios (%)	1.9	4	6.1
Hyperthyroidism (%)	1.9	0.5	0
Hypothyroidism (%)	0	2	0
Vaginitis (%)	1.9	1	0
NICU (%)‡‡	40^b^	10.9^ab^	21.5^a^
Neonatal jaundice (%)‡‡	20	10.9	10
Neonatal deformity (%)‡‡	2.2	1.1	0
Stillbirth (%)‡‡	6.7	0.5^b^	6.2
Preterm birth (%)§§	36.5	12.8^ab^	31.7
Neonatal hypoglycemia (%)‡‡	0	0	0.8
Neonatal asphyxia (%)‡‡	0	5.5	6.2
Neonatal infection (%)‡‡	0	1.1	0

†The values are based on 9 women in group DM, 154 women in group mGDM and 88 women in group sGDM.

‡The values are based on 5 women in group DM, 171 women in group mGDM and 52 women in group sGDM.

§The values are based on 7 women in group DM, 174 women in group mGDM and 77 women in group sGDM.

¶The values are based on 46 women in group DM, 176 women in group mGDM and 106 women in group sGDM.

||The values are based on 31 women in group DM, 164 women in group mGDM and 94 women in group sGDM.

††The values are based on 50 women in group DM, 183 women in group mGDM and 128 women in group sGDM.

‡‡The values are based on 45 women in group DM, 183 women in group mGDM and 130 women in group sGDM.

§§The values are based on 52 women in group DM, 196 women in group mGDM and 126 women in group sGDM.

^a^compare with group DM, there is a significant difference (*P* < 0.05, *P*_adjust_ < 0.017 for multiple comparisons in chi-square test and Kruskal Wallis Test).

^b^compare with group sGDM, there is a significant difference (*P* < 0.05, *P*_adjust_ < 0.017 for multiple comparisons in chi-square test and Kruskal Wallis Test).

OGTT: oral glucose tolerance test; PIH: pregnancy induced hypertension; NICU: neonatal intensive care unit.

Of all patients in the DM group, 37.7% had no treatment during pregnancy, 5.7% only used dietary and 56.6% added insulin. The proportions were respectively 29.3%, 58.6%, 12.1% in mGDM group and 32.6%, 22.7%, 44.7% in sGDM group. Patients adding insulin were significantly fewer in mGDM group than in DM group (*P* < 0.001) and sGDM group (*P* < 0.001). However, in the aspect of treatment, there was no significant difference between DM and sGDM groups (*P* = 0.143). The average blood glucose levels for the patients with various treatments were in Table 2.

**Table 2:** Glycemic levels in different kinds of treatments

	**No treatment**	**Diet only**	**Adding insulin**
OGTT 0 h (mmol/L)†	6.71 ± 2.34	5.68 ± 1.45	7.09 ± 2.52
OGTT 1 h (mmol/L)†	10.93 ± 2.13	10.82 ± 1.84	12.76 ± 2.70
OGTT 2 h (mmol/L)†	10.53 ± 2.92	9.70 ± 2.04	12.80 ± 4.30
Antenatal random glycemia (mmol/L)‡	7.83 ± 3.77	6.08 ± 1.86	7.38 ± 3.42

†The values were based on the data in mGDM and sGDM groups.

‡The values were based on the data in DM, mGDM and sGDM groups.

There was a negative relationship between treatment and several maternal-fetal complications including PIH (OR = 0.57, 95%CI [0.33, 0.99], *P* = 0.047), polyhydramnios (OR = 0.25, 95%CI [0.07, 0.88], *P* = 0.031) and stillbirth (OR = 0.22, 95%CI [0.06, 0.74], *P* = 0.015). Maternal weight before delivery was closely related to PIH (OR = 1.02, 95%CI [1.00, 1.04], *P* = 0.032) and preeclampsia (OR = 1.04, 95%CI [1.01, 1.06], *P* = 0.003). The values of OGTT were not associated with maternal or neonatal complications except PIH, preterm birth, or stillbirth. PIH and preterm birth were closely related to the values of OGTT 0 h. Stillbirth was closely related to the values of OGTT 0 h and 2 h. Of all the neonatal complications, antenatal random glycemia level was mainly related to stillbirth and preterm birth. Table 3 showed the results of logistic regression analysis for blood glucose levels and maternal-fetal outcomes.

**Table 3:** Logistic regression analysis results of blood glucose with the maternal-fetal outcomes

	**PIH**		**Preeclampsia**		**Preterm birth**		**Stillbirth**	
	**Unadjusted**	**Adjusted**	**Unadjusted**	**Adjusted**	**Unadjusted**	**Adjusted**	**Unadjusted**	**Adjusted**
Antenatal random glycemia†								
OR	1.06	1.06	1.07	1.10	1.17	1.19	1.48	1.41
95% CI	0.98-1.15	0.96-1.17	0.98-1.17	0.98-1.23	1.07-1.27	1.08-1.31	1.27-1.72	1.17-1.71
*P* Value	0.116	0.214	0.151	0.105	<0.001	<0.001	<0.001	<0.001
OGTT 0 h‡								
OR	1.21	1.24	1.23	1.22	1.26	1.23	1.64	1.55
95% CI	1.06-1.38	1.04-1.46	1.06-1.43	0.99-1.49	1.09-1.46	1.03-1.47	1.25-2.15	1.14-2.10
*P* Value	0.004	0.015	0.006	0.052	0.002	0.025	<0.001	0.005
OGTT 1 h‡								
OR	1.11	1.21	1.05	1.14	1.06	1.10	1.77	0
95% CI	0.95-1.30	0.98-1.51	0.86-1.29	0.87-1.49	0.91-1.24	0.91-1.35	1.16-2.70	0
*P* Value	0.195	0.08	0.628	0.352	0.423	0.323	0.008	0.948
OGTT 2 h‡								
OR	1.05	1.08	1.07	1.08	1.09	1.07	1.21	1.49
95% CI	0.95-1.16	0.93-1.26	0.95-1.20	0.91-1.27	0.99-1.20	0.95-1.22	1.03-1.42	1.04-2.14
*P* Value	0.35	0.32	0.255	0.384	0.052	0.274	0.022	0.031

†The values were based on the data in DM, mGDM and sGDM groups. The confounders for which OR values were adjusted comprised of age, family history, history of spontaneous abortion, history of stillbirth, living place, maternal weight before delivery and treatment.

‡The values were based on the data in mGDM and sGDM groups. The confounders for which OR values were adjusted comprised of age, family history, history of spontaneous abortion, history of stillbirth, living place, maternal weight before delivery, diagnose time of GDM and treatment.

OGTT: oral glucose tolerance test; PIH: pregnancy induced hypertension.

**Figure 1:**
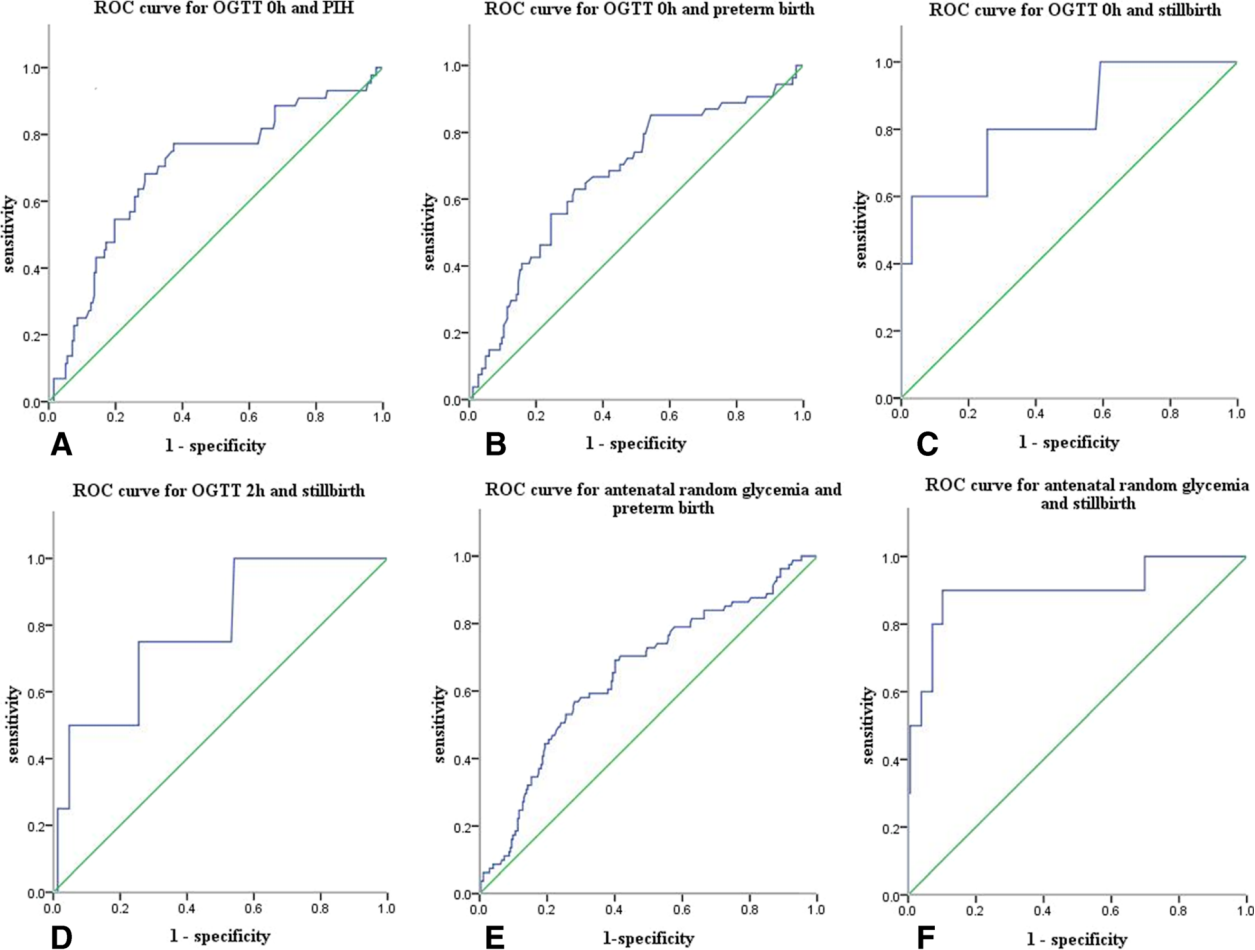
**ROC analysis for hyperglycemia in middle and late pregnancy and maternal-fetal outcomes. (A) **ROC analysis for OGTT 0 h and PIH (*P* < 0.001, areas under the ROC curve: 0.697; sensitivity: 77.3% and specificity: 62.6% for a cutoff of 5.76 mmol/L). **(B)** ROC analysis for OGTT 0 h and preterm birth (*P* < 0.001, areas under the ROC curve: 0.671; sensitivity: 63% and specificity: 68.5% for a cutoff of 5.96 mmol/L). **(C)** ROC analysis for OGTT 0 h and stillbirth (*P* = 0.013, areas under the ROC curve: 0.826; sensitivity: 60% and specificity: 96.9% for a cutoff of 11.78 mmol/L). **(D)** ROC analysis for OGTT 2 h and stillbirth (*P* = 0.049, areas under the ROC curve: 0.787; sensitivity: 75% and specificity: 74.4% for a cutoff of 11.7 mmol/L). **(E)** ROC analysis for antenatal random glycemia and preterm birth (*P* < 0.001, areas under the ROC curve: 0.653; sensitivity: 69.1% and specificity: 59.9% for a cutoff of 6.30 mmol/L). **(F)** ROC analysis for antenatal random glycemia and stillbirth (*P* < 0.001, areas under the ROC curve: 0.901; sensitivity: 90% and specificity: 89.9% for a cutoff of 10 mmol/L).

ROC analysis showed that the value of OGTT 0 h could predict PIH in this population (*P* < 0.001). Both the values of OGTT 0 h and antenatal random glycemia could predict preterm birth (*P* < 0.001). For stillbirth, the values of OGTT 0 h, OGTT 2 h and antenatal random glycemia were predictors (*P* = 0.013, *P* = 0.049, *P* < 0.001). Details were shown in Figure 1.

## Discussion

From the results, we found that the maternal basic characters, maternal complications and neonatal complications except NICU were similar in sGDM group and DM group. This suggests that GDM patients with fasting glucose ≥ 7 mmol/L or OGTT 2 h ≥ 11.1 mmol/L might have diabetes before pregnancy. The prognoses of the two groups were worse than that of the mGDM group, especially the sGDM group. This might result from the later diagnosis time of sGDM patients, so far as to ketoacidosis appearing. Most patients in sGDM group were diagnosed in the 33rd week of pregnancy who might have suffered glycemic abnormality for a long period without treatment and have developed some maternal complications in an early phase, let alone the influence of combination with poor treatments. In this study, for all patients together, the average antenatal random glycemia of patients adding insulin was 7.38 ± 3.42 mmol/L which did not satisfy the control standards and was much higher than that of patients only using diet. This indicates that the added dose of insulin might be not enough to control the blood sugar. Many Chinese women are reluctant to use medicine during pregnancy because of the misconceptions that the medicines used might harm their babies. Even if some patients in DM group had received insulin therapy before pregnancy, they refused insulin injection after they became pregnant. The bad relationship between doctors and patients in China might also have contributed to the inadequate usage of insulin. Some pregnant women didn’t regularly come to do prenatal examinations or monitor blood glucose at home, even doubted what doctors said. Moreover, compliance to the usage of insulin in sGDM group was worse than those patients with diabetes for many years for that they had never come into contact with insulin injection before, so their treatment efficiency was even worse than that in DM group. Some random control trials have demonstrated that for gestational diabetes, a comprehensive management of dietary and necessary insulin could significantly improve the perinatal outcomes [28,29]. Therefore, we should emphasize health education and help those women change the misunderstanding of medicine use in pregnancy. Moreover, we should pay more attention to the patients with sGDM such as adding frequency of antenatal care, closely monitoring in clinical work and the most important one is early diagnosis.

In addition, more patients in DM and sGDM groups required insulin supplement. The average blood glucose levels of OGTT of those adding insulin could be used as references for clinicians, with the aim of managing the glycemia in an earlier stage and avoiding severe maternal and neonatal complications.

One study has suggested that, in GDM, increased severity of insulin resistance and related features of the “metabolic syndrome” are precursors to the development of preeclampsia [30]. Maternal obesity could aggravate the complications [31]. In our study, maternal weight before delivery was closely positively related to PIH and preeclampsia in accordance with the studies above. What’s more, through the ROC analysis, the value of OGTT 0 h could predict the occurrences of PIH and preterm birth; the values of OGTT 0 h and OGTT 2 h could both predict the occurrence of stillbirth. Therefore, we should pay more emphasis on those with OGTT 0 h ≥ 5.76 mmol/L or OGTT 2 h ≥ 11.7 mmol/L, at the same time actively control the blood glucose under the target (fasting < 5.3 mmol/L, postprandial 2 h < 6.7 mmol/L) and rigorously monitor blood pressure, maternal weight, fetal heart rate and fetal movement so as to reduce severe maternal and neonatal complications.

Glycemic level in the late pregnancy is directly related to the baby’s safety. Even in women with a mild degree of GDM, proper management of both mother and fetus could reduce the number of unexplained stillbirths [32]. Our study also showed that women with poor perinatal blood sugar easily suffered stillbirth and premature birth. Premature birth might lead to neonatal long term complications which might bring economic burden to both family and society. Therefore, active control of antenatal blood glucose level is an effective method to prevent neonatal complications.

However, there were some limitations in our research. It was a single center study and the sample size was small, so the sensitivity and specificity of results were not so satisfactory. With the standardization of the diagnosis and treatment of GDM, we will get more accurate and particular data in China.

## Conclusions

Pregnant women in the mGDM group have better outcomes than those in the DM and sGDM groups. The values of OGTT and antenatal random glycemia could predict PIH, stillbirth or preterm birth to some extent. Active control of antenatal blood sugar is beneficial to reduce neonatal complications. What’s more, for the pregnant women with OGTT 0 h ≥ 7 mmol/L or OGTT 2 h ≥ 11.1 mmol/L, we should actively use enough insulin, perform health education and improve maternal compliance so as to avoid severe maternal-fetal complications.

## Competing interests

The authors declare that they have no competing interests.

## Authors’ contributions

JG involved in conception of the research; collected materials and disposed data; carried out statistic analysis; drafted the article. AL collected materials and disposed data. XS collected materials and disposed data. LF provided guidance for the design and revised the manuscript. All authors read and approved the final manuscript.

## Pre-publication history

The pre-publication history for this paper can be accessed here:

http://www.biomedcentral.com/1471-2393/14/34/prepub
